# Fingerspelling as a Novel Gateway into Reading Fluency in Deaf Bilinguals

**DOI:** 10.1371/journal.pone.0139610

**Published:** 2015-10-01

**Authors:** Adam Stone, Geo Kartheiser, Peter C. Hauser, Laura-Ann Petitto, Thomas E. Allen

**Affiliations:** 1 Educational Neuroscience (PEN) Program, Gallaudet University, Washington, DC, United States of America; 2 NSF Science of Learning Center on Visual Language and Visual Learning (VL2), Gallaudet University, Washington, DC, United States of America; 3 Deaf Studies Laboratory, National Technical Institute for the Deaf, Rochester Institute of Technology, Rochester, New York, United States of America; 4 Department of Psychology, Gallaudet University, Washington, DC, United States of America; 5 Department of Education, Gallaudet University, Washington, DC, United States of America; Birkbeck College, UNITED KINGDOM

## Abstract

Studies have shown that American Sign Language (ASL) fluency has a positive impact on deaf individuals’ English reading, but the cognitive and cross-linguistic mechanisms permitting the mapping of a visual-manual language onto a sound-based language have yet to be elucidated. Fingerspelling, which represents English orthography with 26 distinct hand configurations, is an integral part of ASL and has been suggested to provide deaf bilinguals with important cross-linguistic links between sign language and orthography. Using a hierarchical multiple regression analysis, this study examined the relationship of age of ASL exposure, ASL fluency, and fingerspelling skill on reading fluency in deaf college-age bilinguals. After controlling for ASL fluency, fingerspelling skill significantly predicted reading fluency, revealing for the first-time that fingerspelling, above and beyond ASL skills, contributes to reading fluency in deaf bilinguals. We suggest that both fingerspelling—in the visual-manual modality—and reading—in the visual-orthographic modality—are mutually facilitating because they share common underlying cognitive capacities of word decoding accuracy and automaticity of word recognition. The findings provide support for the hypothesis that the development of English reading proficiency may be facilitated through strengthening of the relationship among fingerspelling, sign language, and orthographic decoding en route to reading mastery, and may also reveal optimal approaches for reading instruction for deaf and hard of hearing children.

## Introduction

What are the factors contributing to English reading fluency in deaf and hard of hearing people who primarily use American Sign Language (ASL), a soundless, natural language with few obvious phonological or orthographic links to English? A novel aim of the present work is to identify these factors so that we may begin to consider how best to facilitate their acquisition in young deaf children. Reading fluency is defined as the ability to read text with speed and accuracy, with fluent readers being able to decode and comprehend text simultaneously [1, 2]. The National Reading Panel has acknowledged fluency to be one of the five critical components of skilled reading, along with phonemic awareness, phonics, vocabulary, and text comprehension [3]. Dysfluent readers are prone to making many errors and their reading may be slow and laborious as they struggle to identify words. When reading aloud, they may sound monotonic, halting, and unnatural, lacking proper expression.

Two core elements make up reading fluency: accuracy of word decoding, and automaticity of word recognition [4]. Accuracy of decoding refers to the ability to correctly identify the word, either via one’s sight-word vocabulary, or via decoding strategies such as sounding out the word or analyzing it for roots and affixes. Next, the reader needs to quickly recognize the word and move to the next word with little mental exertion, so that cognitive resources are available for other tasks such as passage comprehension or reading aloud with good prosody. Some have suggested that good oral prosody represents a third core element in defining reading fluency, but it may also be an outcome of utilizing the first two core elements of accuracy and automaticity [4].

Crucially, fluency has been linked with reading comprehension. Measures of reading fluency have found to be better predictors of reading comprehension than other measures (e.g., phonological awareness), and fluent readers are more likely to score higher on reading comprehension assessments [5, 6]. Poor readers have been shown to improve their reading comprehension skills when provided with fluency instruction [7]. The National Reading Panel [3] expressed concern that children’s reading difficulties may be attributed in part to their fluency skills, and that this topic remained severely understudied compared to other areas of reading comprehension.

Similarly there exists a lack of empirical research in the fluency of readers who are deaf or hard of hearing [8]. A synthesis of peer-reviewed research in reading fluency in English in this population revealed only six studies published between 1970 and 2009 [9]. Four studies used fluency-based educational interventions (i.e. improving automaticity of word recognition) for supporting reading skills in deaf and hard of hearing students. All reported positive gains [10, 11, 12, 13], while a causal-comparative study found that skilled deaf adolescent readers read faster than average or below-average readers [14]. The sixth study used a correlational design comparing word and reading comprehension scores with a rubric measuring signed reading fluency (i.e., how fluently they signed aloud English text) in young and adolescent deaf readers; as signed reading fluency scores increased, so did comprehension scores [8]. However, none of these studies measured participants’ linguistic competence in, and comprehension of, American Sign Language (ASL), a naturally-evolved visual signed language used by deaf communities and schools in the United States and parts of Canada; as will be discussed shortly, measuring children’s linguistic competences in ASL (or a signed language) may be crucial to better understanding the overall picture of reading capabilities in this population.

Studies of reading proficiency in profoundly deaf children and adults have traditionally examined their sound-based phonological awareness and sound decoding skills of English letters on the page instead of fluency. Mayberry, del Giudice, and Lieberman [15] in a meta-analysis of 57 studies involving deaf readers, found that sound phonological awareness and sound phonological decoding skills did not strongly predict reading proficiency in deaf individuals, unlike what is commonly found in hearing readers. Instead, language ability emerged as the strongest predictor of reading proficiency. Specifically, sign language proficiency has been consistently linked with higher reading comprehension in deaf readers [16–22], with the age of first bilingual exposure, in this case, ASL and English, being a strong predictor of bilingual language and reading mastery [23, 24, 25]. However, researchers and educators have struggled to articulate a sound, testable model describing how proficiency in a signed (soundless) language translates into proficiency with decoding and comprehending the orthography of a second, unrelated, and sound-based language. In short, there remains an unexplained “missing link” between signed language and written language.

Padden and Hanson [26] have suggested that fingerspelling—the use of hands and fingers to symbolize written orthography—represents that “missing link.” Fingerspelling has been part of signed languages for centuries, dating back to the early 17^th^ century when educators noted its potential application to facilitating reading instruction in deaf children [27, 28]. In ASL, both the nature and degree of use of fingerspelling in a typical conversation will differ based on a continuum of conventionalization [29–33]. At one end is serial fingerspelling. Here, signers produce a linear string of hand configurations that represent individual letters of the English alphabet and have little or no sign-phonological assimilation among the hand configurations (as per the segmental and combinatorial linguistic rules of ASL phonological structure). At the other end is lexicalized fingerspelling. Here, a fingerspelled sequence has undergone extensive sign phonological changes such that it is conventionalized and is formed with the same rhythmic-temporal patterning key to sign-phonetic and sign-syllabic linguistic organization of ASL signs. In effect, the fingerspelled form assumes the typical linguistic properties of a lexicalized loan word and functions like a stable lexical item in the language.

In addition, fingerspelling appears to be acquired naturally by children with signing deaf parents and children perceive and produce fingerspelling at regular linguistic developmental milestones [32]. Remarkably, fingerspelling undergoes similar universal stages of language acquisition, whereupon young signers first learn a fingerspelled unit’s overall form (as if it were a stable entry in their lexicon), and only later decompose it. This is similar to, for example, the hearing English child’s early acquisition of “wanna” as a stable lexical form, and then later correctly decompose it into the verb “want” and preposition “to.”) Specifically, young signers learn and produce only the lexicalized variety of fingerspelling first, attending primarily to its overall movement envelope (ages 0–4 years) [29]. Then, at ages 4–6 years, as they begin learning the alphabetic principle, they now attend to and analyze the internal componential units of the fingerspelled form, the individual letters. It is only in this later stage that children realize that fingerspelling is composed of individual hand configurations corresponding directly to English letters. From this point on, the child then uses both varieties of fingerspelling according to stable morphological and lexical rules in signed language governing when and how to use fingerspelling.

Educators of deaf and hard of hearing children have noted the utility of fingerspelling for reading instruction, due to its direct 1:1 relationship between handshapes and English letters, and have devised ways to use fingerspelling systematically (using sequences called “chaining” and “sandwiching”) when introducing new English vocabulary [34]. The incorporation of both serial and lexicalized varieties of fingerspelling in elementary reading instruction has been demonstrated to enhance English vocabulary acquisition in deaf children [35, 36, 37]. Confirming educators’ intuitions, empirical studies have found a relationship between reading skills and fingerspelling in deaf children and adults. Allen [38] demonstrated that fingerspelling abilities of deaf children as young as age 4, in combination with their ASL receptive skills, predicted their ability to write letters of the alphabet. Padden and Hanson [26] compared fingerspelling and reading performances in deaf readers ages 8 to 14 and found correlations between the two (*r* = .50 to .71). In a study of how adult deaf readers segment print and fingerspelled words, Emmorey and Petrich [39] discovered a medium-size correlation between fingerspelling ability and reading scores (*r* = .35). Most recently and using the NSF Science of Learning Center, Visual Language & Visual Learning (VL2) Psychometric Toolkit dataset, Morere and Koo [40] also found the same relationship between fingerspelling and reading comprehension (*r* = .61).

What could account for this relationship between fingerspelling and reading comprehension in deaf children and adults? Padden and Hanson [26] offer clues regarding automaticity and accuracy of word recognition, which are key capacities in reading fluency [4]. In this study, deaf children, ages 8–14, could identify a fingerspelled English word correctly about 95% of the time after viewing it just once, which is a remarkable indication of how skillful deaf singing children are at perceiving rapidly-presented strings of fingerspelled letters. Moreover, the youngest children made more errors than did older children in writing down fingerspelling sequences they had just seen, but only if the fingerspelled sequences were low-frequency words—they performed better with high-frequency words. Given that the youngest children also have the least experience with reading, this suggests that the younger children are learning the segmental and distributional probabilities of English letter combinations and using this statistical information to accurately decode fingerspelled words. This suggests that accuracy of word decoding—a key capacity in reading fluency—also plays a factor in comprehending fingerspelling. In addition, those fingerspelled words were not presented in isolation but were inserted into signed sentences requiring another key reading fluency capacity: automaticity of word recognition. Without automaticity, the child may get stuck trying to decode and identify the fingerspelled word, ignoring or missing the rest of the sentence as it was being signed. However, this did not happen in Padden and Hanson’s sample [26] as all children had a low error rates in selecting a correct illustration (out of 4) to match the sentence, indicating they were able to automatically decode the word and attend to the rest of the sentence as it was being signed. In all, the children in this study demonstrated a high level of skill with fingerspelling, and were able to quickly and accurately decode fingerspelled sequences within whole, signed sentences even at early ages.

We know that fingerspelling involves a rapidly presented sequence of hand configurations intended to be perceived and decoded quickly and accurately, and that these sequences of hand configurations are direct representations of English words and letters. We also know that accuracy of word decoding and automaticity of word recognition are both vital precursors to reading fluency [4, 41]. Given that young deaf children have experience with understanding fingerspelling long before they begin reading, skilled fingerspelling and its visual-manual representation of English letter combinations may support accuracy of word decoding and automaticity of word recognition, which, in turn, enhances reading fluency and the acquisition of skilled reading. Here, we hypothesize that deaf readers who are skilled at fingerspelling will also demonstrate greater fluency in reading due to shared underlying cognitive capacities involving word decoding accuracy and word recognition automaticity [16–25].

## Methods

### Participants

As part of the VL2 Toolkit Psychometric Study [42], a sample of 32 deaf individuals from Gallaudet University and the Washington, D.C. metropolitan area completed assessments of fingerspelling skills and reading fluency. One outlier (defined as scoring 2.5 standard deviations above or below on any assessment measure) was removed, resulting in a final sample of 31 (18 females, 11 males, 2 did not report gender). Participants ranged in age from 19 to 44 years (*M* = 25.9, *SD* = 7.37). The ethnic distribution was comparable to that of the larger university population: 48.4% European American, 22.7% African American, 9.7% Asian American, 16.1% Latino/Hispanic, and 16.1% Native American, 6.5% Other (participants could select more than one). All were undergraduate students. This sample also had variability in hearing status and language experience: 64.5% were born deaf or hard of hearing, 72.4% reported having severe to profound hearing loss, and 19.4% reported having at least one deaf parent. 38.7% of participants were exposed to ASL at birth or before five years of age (commonly known as “native signers”). 50.0% of participants reported wearing hearing aids on a regular or occasional basis, and one participant reported using a cochlear implant on a regular basis.

The Institutional Review Board at Gallaudet University approved this study. All participants were provided with an explanation of the project and explanation of informed consent in both written English and ASL, and participants indicated their consent in writing. Participation was also strictly voluntary and participants were informed that they could withdraw at any time without penalty.

### Procedures

Participants were first given a link to the VL2 Background Questionnaire to complete using a computer. Next, an assessment battery was conducted in three sessions, each approximately 2.5–3 hours long, and occurring across at least three days. Assessments were counterbalanced across sessions [43]. Approximate times for the K-BIT, ASL-SRT, the VL2 Fingerspelling Test, and the WJ-III Reading Fluency test were 20, 15, 30, and 3 minutes, respectively. Participants received compensation after completion of each testing session. All data was anonymized with participant IDs to preserve confidentiality, then entered into a database.

### Measures

#### VL2 Background Questionnaire

The VL2 Background Questionnaire is composed of 101 questions related to participants’ background characteristics and demographics, including deafness and language/assistive device usage, parent and family characteristics, language history, educational history, and relevant medical information (e.g. visual acuity; related hearing disorders; neurological, vestibular, and learning conditions) [42]. This questionnaire was administered online via a weblink provided to participants prior to the assessment session.

#### Kaufman Brief Intelligence Test

Overall cognitive functioning was estimated via administering the Matrices subtest of the Kaufman Brief Intelligence Test, 2^nd^ Edition (KBIT-2) [44]. The Matrices subtest specifically uses visuospatial stimuli, thus eliminating the impact of language on the participant’s performance. Raw scores were used in this study; however, the mean standardized score of all participants in the VL2 Psychometric Toolkit Study was consistent with test norms and reflected expectations of cognitive functioning in a college population (*n* = 48, *M* = 104.48, *SD* = 10.96) [45].

#### Corsi Block Task

Working memory, the ability to hold and manipulate cognitive representations, has been shown to have important effects on the performance of many different cognitive tasks, including reading [46, 47]. Based on that knowledge, it is reasonable to speculate that deaf individuals’ reading abilities may be determined in part by their working memory capacity. More specifically, it is possible that deaf individuals’ performance on the VL2 Fingerspelling Test (described below) may be linked to their ability to remember and manipulate sequences of handshapes representing alphabetic letters. To examine this possibility, we included a measure of working memory in our statistical model to account for its possible confounding effects on reading fluency. Verbal working memory is a good predictor of reading ability in hearing individuals [48]. However, because our primary independent variables of interest (fingerspelling and ASL ability) are presented in a visual-spatial modality, we have elected to include a measure that taps into the participants’ visual and spatial working memory abilities. Typically, measures of linguistic working memory among signers employ sequences of fingerspelled letters. Thus, these typical measures are inherently confounded with our primary variable of interest. Using a measure of non-linguistic, visual-spatial working memory avoids this confounding and provides an independent measure of working memory.

Working memory was measured via administering the backward Corsi block task, where the individual is asked to repeat, in reverse order, a sequence of locations on a randomly distributed array of blocks [49]. Here, the stimulus was a square with an array of nine raised cubes. The examiner touches the blocks in a sequence, one block per second, and the participant is asked to repeat the sequence in reverse order. A series of pattern sets was used, in which the sets increased in length, starting with a two-item set up to a maximum set of nine blocks. No block is indicated twice within a set. Each set has two trials, and the task continues until the participant makes an error on both trials in a set or until all of the sets are completed. Prior analyses revealed working memory as measured on this task to correlate with general reading comprehension (not fluency) measured using the Passage Comprehension subtest of the Woodcock-Johnson III Tests of Achievement, *r* = 0.29, *p* < 0.05 [50].

#### ASL Sentence Reproduction Test

Each participant’s ASL proficiency was assessed using the ASL Sentence Reproduction Test, a standardized test [51]. The ASL-SRT presents participants with 20 ASL sentences signed by a native signer. The participant is asked to view each sentence and then reproduce the sentence exactly as it was signed. The sentences increase in syntactic, thematic, and morphemic complexity. Trained raters evaluated each sentence: if the sentence was reproduced exactly as signed, it was rated as 1; if one or more errors occurred, it was rated as 0. The raw score is calculated by totaling the number of sentences reproduced exactly as signed, and ranges from 0 (no correct reproductions) to 20 (all sentences reproduced exactly as signed). Inter-rater reliability of the ASL-SRT was reported to be *r* = 0.83, *p* < 0.01 [51], and prior analyses revealed achievement on the ASL-SRT to correlate with general reading comprehension (not fluency) measured using the Passage Comprehension subtest of the Woodcock-Johnson III Tests of Achievement, *r* = 0.48, *p* < 0.001 [18].

#### VL2 Fingerspelling Test

The VL2 Fingerspelling Test was developed as part of the VL2 Psychometric Toolkit [52] and uses the same real word and pseudoword lists from the Spelling and Spelling of Sounds subtests of the Woodcock-Johnson III Test of Achievement [53]. A total of 45 real words and 25 psuedowords were mixed, beginning with easier words and increasing in difficulty as the test progressed. Participants watched fingerspelled words on video and were instructed to fingerspell (while being videotaped) what they had just seen. The raw score is the number of correct responses. Prior analyses have found that fingerspelling proficiency correlates significantly with general reading comprehension (not fluency), measured using the Peabody Individual Achievement Test (PIAT), *r* = 0.61, *p* < 0.01, and with ASL proficiency, measured using the ASL-SRT, *r* = 0.52, *p* < 0.01 [52].

#### Woodcock-Johnson III Reading Fluency

The Woodcock-Johnson (WJ-III) Reading Fluency subtest [54] was used to evaluate participants’ English reading fluency by looking specifically at their reading speed and semantic processing speed. The subtest requires each participant to read many short sentences rapidly and respond to each sentence by circling “true” or “false,” all within a 3-min period. The raw score is the number of correct responses. In deaf individuals, achievement on this assessment correlates significantly with both general reading comprehension measured using the PIAT, *r* = 0.68, *p* < 0.01, and ASL proficiency measured using the ASL-SRT, *r* = 0.45, *p* < 0.01 [53].

## Results

The means, standard deviations, and inter-correlations of the three predictor variables of interest (Age of ASL Acquisition, ASL Proficiency, and Fingerspelling Skill), the two covariates (Cognitive Functioning, Working Memory), and the one outcome variable (Reading Fluency) are presented in Table 1. The three predictor variables were highly correlated with each other, but high tolerance values of .803, .643, and .692, and correspondingly low variance inflation factors (VIFs) of 1.246, 1.555, and 1.446 indicated a small and acceptable degree of multicollinearity among them.

**Table 1:** Means, standard deviations, and inter-correlations of predictor and outcome variables.

Variable	*M*	*SD*	1	2	3	4	5	6
1. Cognitive Functioning (KBIT-2)	37.87	3.35	–	.42*	-.24	.43*	.35	.14
2. Working Memory (Corsi Block)	5.77	.99		–	-.35	.29	.35	.19
3. Age of ASL Acquisition	9.00	6.61			–	-.39*	-.38*	-.43*
4. ASL Fluency (ASL-SRT)	8.74	4.37				–	.50**	.57***
5. Fingerspelling Skill	51.23	9.22					–	.67***
6. Reading Fluency (WJ-III)	70.03	22.53						–

* *p* < .05 (two-tailed)

** *p* < .01 (two-tailed)

*** *p* < .001 (two-tailed)

The hypothesis was tested using a hierarchical multiple regression analysis. To investigate whether fingerspelling skill predicted reading fluency apart from age of ASL acquisition and general ASL linguistic proficiencies while controlling for cognitive functioning and working memory, the analysis was conducted by entering cognitive functioning, working memory, and age of ASL acquisition in the first step, ASL proficiency in the second step, followed by fingerspelling skill in the third step. The results are presented in Table 2. After controlling for cognitive functioning, the hierarchical multiple regression analysis found fingerspelling skill to significantly predict reading fluency (∆*R* ^*2*^ = .19, ∆*F* (5, 25) = 11.65, *p* = .002, β = .53) over and above age of ASL acquisition and ASL proficiency. Together, the three predictors described 59% of the variance in reading fluency scores.

**Table 2:** Summary of hierarchical multiple regression analysis for age of ASL acquisition, ASL fluency, and fingerspelling skill predicting reading fluency scores.

Predictor and Step	β	R^2^	∆R^2^	∆F†
Step 1				
Cognitive Functioning	.02	.19	.19	2.11
Working Memory	.04			(3, 27)
Age of ASL Acquisition	-.41*			
Step 2				
Cognitive Functioning	-.16	.40	.21	9.44**
Working Memory	.02			(4, 26)
Age of ASL Acquisition	-.26			
ASL Proficiency	.54**			
Step 3				
Cognitive Functioning	-.22	.59	.19	11.65**
Working Memory	-.06			(5, 25)
Age of ASL Acquisition	-.17			
ASL Proficiency	.35*			
Fingerspelling Skill	.53**			

* *p* < .05 (two-tailed)

** *p* < .01 (two-tailed)

† *Degrees of freedom are indicated below the F-value*.

## Discussion

The goal of this study was to explore how fingerspelling may represent a “missing link” between signed language and skilled reading in deaf and hard-of-hearing people. Specifically, we asked whether deaf readers who are skilled at fingerspelling would also demonstrate greater reading fluency due to shared underlying capacities of word decoding accuracy and word recognition automaticity. We performed a hierarchical multiple regression using cognitive functioning, working memory, age of ASL acquisition, ASL proficiency, and fingerspelling skill as predictor variables of reading fluency, and found that fingerspelling skill significantly predicted reading fluency over and above age of ASL acquisition and ASL proficiency. In addition, we also verified that, within a model incorporating fingerspelling skill and linguistic competence, working memory did not play a significant role in predicting reading fluency.

This finding corroborates past discoveries of relationships between fingerspelling and reading skill [26, 38, 39, 40]. However, the novel contribution is the presentation of findings that suggest that the rapid and accurate decoding of fingerspelled words predicts the rapid and accurate decoding of printed words, suggesting a common underlying decoding, or fluency, skill that develops in different modalities. Young signing deaf children learn and use fingerspelling years before they begin reading, and the perception and decoding of fingerspelling appears to draw on both accuracy of word decoding and automaticity of word recognition [26]. It is these years of experience with understanding fingerspelling before they begin to read which may strengthen their word decoding accuracy and word recognition automaticity and also increases their exposure to statistical regularities of English letter combinations [55]. When deaf children begin to learn how to read at around age 5, these strengths, derived from their prior experience with their visual-manual language, inclusive of fingerspelling, may lead to an early literacy advantage for deaf signing children resulting in earlier and more robust achievement of reading fluency and an overall enhancement in the acquisition of skilled reading.

We note the fascinating finding that fingerspelling skill predicts reading fluency over and above ASL proficiency alone, suggesting that while general signed language proficiency and fingerspelling skill jointly contribute to reading skill, both do so separately via different cognitive and literacy mechanisms. This explanation may account for why some participants in this sample demonstrated high ASL proficiency, but low fingerspelling receptive skill. While the importance of early visual language exposure for deaf children has been well established [21–25], the divergence in general sign language proficiency and specific fingerspelling skill suggests an area of exciting future research where the relative weighting of fingerspelling and early exposure to sign in producing skilled readers can be further studied. One theoretical model that may be of use here is the Simple View of Reading which hypothesizes that reading comprehension is composed of two components: decoding and linguistic competence [56, 57]. Here, decoding is defined as the ability to see a word in print, rapidly access the mental lexicon, and retrieve the meaning, while linguistic competence refers to the ability to understand language (for example, being able to accurately answer comprehension questions after listening to a story narrative). In this model, both components are related to reading comprehension in a multiplicative relationship (R = D × L) such that both skills must be present at sufficiently high levels in order to produce high reading comprehension abilities.

Chamberlain and Mayberry [58] have proposed that the Simple View of Reading model is applicable to deaf signing readers and that fingerspelling represents an important element in the decoding component by emphasizing principled relationships between orthographic patterns and the sublexical, phonological structure of sign language and supporting rapid, automatic, and accurate word recognition [26]. Indeed, in the final hierarchical regression model, the two largest contributors to reading fluency were fingerspelling skill and ASL proficiency, which could be broadly construed to represent the decoding and linguistic competence components, respectively. Our results’ alignment with the Simple View of Reading as a potential theoretical model further adds credence to our novel hypothesis that fingerspelling may be a crucial component in the development of reading in young deaf signing children due to shared underlying constructs of word decoding accuracy and word recognition automaticity and increased exposure to English orthographic patterning. Recently, Williams, Darcy, and Newman [59] examined fingerspelling priming effects in deaf readers and found that deaf readers demonstrated a faster response time for fingerspelled and print words sharing orthographic similarities than for those sharing sound phonological similarities, suggesting again that both fingerspelling and print recognition is supported by common cognitive processes centering around processing visual orthographic information.

The question of what parts of signed language—a soundless language—contribute to reading the orthography of spoken languages has been one of the most passionately explored areas in reading research. We hope that our work contributes to a better understanding of one of the parts: specifically, how early experience with fingerspelling influences the acquisition of skilled reading. The results suggest that deaf children with early and frequent exposure to fingerspelling may be better prepared to crack the code of reading when they enter school. These results further confirm common intuitions held by well-trained educators who have long recognized the importance of fingerspelling in reading instruction and who have already developed specific English vocabulary instruction methods that incorporate fingerspelling [34–37]. Building on these existing capacities may help us better develop optimal environments targeted to enhance learning to read in young deaf and hard of hearing children. Equally exciting, novel reading interventions can be designed using principles of fingerspelling and visual language for at-risk hearing readers who demonstrate symptoms of dysfluent reading. That is, all children, deaf and hearing alike, stand to benefit from a better understanding of how fingerspelling, by promoting accuracy and automaticity of word recognition, contributes to reading and how learning to read—which takes place primarily in the visual modality—can be enhanced by using elements of visual signed language, with the concurrent revelation that access to sound is *not* obligatory for achieving reading success.

## Supporting Information

S1 Data A zipped file containing the fingerspelling and fluency dataset (in SPSS format) used for this data analysis and a codebook (in PDF format). (ZIP)
