# Supplementary material for: Fingerspelling as a Novel Gateway into Reading Fluency in Deaf Bilinguals
Source: PLoS One. 2015 Oct 1;10(10):e0139610. doi: 10.1371/journal.pone.0139610 (PMC4591273; doi:10.1371/journal.pone.0139610)
Supplement: S1 Data — (ZIP) [file pone.0139610.s001.zip › Fingerspelling and Fluency PLoS Data Codebook.pdf]

Codebook

[DataSet3] /Users/adamstone/Dropbox/My Research/Papers/Fingerspelling Fluency Paper/Fingerspelling and Fluency PLoS Data Set.sav

VL2ID

		Value	Count	Percent
Standard Attributes	Position	1		
	Label	VL2 ID		
	Type	Numeric		
	Format	F11		
	Measurement	Nominal		
	Role	Input		
Custom Attributes	$ODBC.Name	VL2_ID		
	$ODBC.Table	Audiological_ITEM_TBL		
	$ODBC.Size	10		
	$ODBC.Type	4		
Valid Values	24		1	3.2%
	26		1	3.2%
	38		1	3.2%
	64		1	3.2%
	67		1	3.2%
	75		1	3.2%
	92		1	3.2%
	102		1	3.2%
	108		1	3.2%
	116		1	3.2%
	150		1	3.2%
	171		1	3.2%
	191		1	3.2%
	214		1	3.2%
	216		1	3.2%
	224		1	3.2%
	244		1	3.2%
	265		1	3.2%
	279		1	3.2%
	290		1	3.2%
	315		1	3.2%
	320		1	3.2%
	321		1	3.2%
	324		1	3.2%
	325		1	3.2%
	349		1	3.2%
	358		1	3.2%
	374		1	3.2%
	387		1	3.2%
	395		1	3.2%
	397		1	3.2%

AgeASL

		Value
**Standard Attributes**	**Position**	2
	**Label**	Age when you stared using ASL?
	**Type**	Numeric
	**Format**	F8
	**Measurement**	Scale
	**Role**	Input
**N**	**Valid**	31
	**Missing**	0
Central Tendency and Dispersion	**Mean**	9.00
	**Standard Deviation**	6.608
	**Percentile 25**	2.00
	**Percentile 50**	9.00
	**Percentile 75**	16.00

RAW_PIATR

		Value
**Standard Attributes**	**Position**	3
	**Label**	PIAT-R Raw Score
	**Type**	Numeric
	**Format**	F11
	**Measurement**	Scale
	**Role**	Input
**Custom Attributes**	**$ODBC.Name**	RAW_PIAT-R
	**$ODBC.Table**	PSS_TBL
	**$ODBC.Size**	10
	**$ODBC.Type**	4
**N**	**Valid**	31
	**Missing**	0
Central Tendency and Dispersion	**Mean**	72.39
	**Standard Deviation**	13.923
	**Percentile 25**	63.00
	**Percentile 50**	74.00
	**Percentile 75**	85.00

RAW_RF_WJ

		Value
**Standard Attributes**	**Position**	**4**
	**Label**	WJ-III Reading Fluency Raw Score
	**Type**	**Numeric**
	**Format**	**F11**
	**Measurement**	**Scale**
	**Role**	**Input**
**Custom Attributes**	**$ODBC.Name**	**RAW_RF_WJ**
	**$ODBC.Table**	**PSS_TBL**
	**$ODBC.Size**	**10**
	**$ODBC.Type**	**4**
**N**	**Valid**	**31**
	**Missing**	**0**
Central Tendency and Dispersion	**Mean**	**70.03**
	**Standard Deviation**	**22.532**
	**Percentile 25**	**54.00**
	**Percentile 50**	**65.00**
	**Percentile 75**	**91.00**

RAW_KBMatrices

		Value
**Standard Attributes**	**Position**	**5**
	**Label**	K-BIT2 Matrices Raw Score
	**Type**	**Numeric**
	**Format**	**F11**
	**Measurement**	**Scale**
	**Role**	**Input**
**Custom Attributes**	**$ODBC.Name**	RAW_KBMatrices
	**$ODBC.Table**	**PSS_TBL**
	**$ODBC.Size**	**10**
	**$ODBC.Type**	**4**
**N**	**Valid**	**31**
	**Missing**	**0**
Central Tendency and Dispersion	**Mean**	**37.87**
	**Standard Deviation**	**3.354**
	**Percentile 25**	**35.00**
	**Percentile 50**	**38.00**
	**Percentile 75**	**40.00**

RAW_ASLSRT

		Value
Standard Attributes	Position	6
	Label	ASL-SRT Total Correct
	Type	Numeric
	Format	F11
	Measurement	Scale
	Role	Input
Custom Attributes	$ODBC.Name	RAW_ASL-SRT
	$ODBC.Table	PSS_TBL
	$ODBC.Size	10
	$ODBC.Type	4
N	Valid	31
	Missing	0
Central Tendency and Dispersion	Mean	8.74
	Standard Deviation	4.366
	Percentile 25	5.00
	Percentile 50	8.00
	Percentile 75	12.00

RAW_TC_FST

		Value
Standard Attributes	Position	7
	Label	Finger Spelling Test Total Correct
	Type	Numeric
	Format	F11
	Measurement	Scale
	Role	Input
Custom Attributes	$ODBC.Name	RAW_TC_FST
	$ODBC.Table	PSS_TBL
	$ODBC.Size	10
	$ODBC.Type	4
N	Valid	31
	Missing	0
Central Tendency and Dispersion	Mean	51.23
	Standard Deviation	9.218
	Percentile 25	44.00
	Percentile 50	52.00
	Percentile 75	60.00

RAW_BSpan_MAN

		Value
**Standard Attributes**	**Position**	8
	**Label**	Corsi Blocks MAN BWD Span
	**Type**	Numeric
	**Format**	F11
	**Measurement**	Scale
	**Role**	Input
**Custom Attributes**	**$ODBC.Name**	RAW_BSpan_MAN
	**$ODBC.Table**	PSS_TBL
	**$ODBC.Size**	10
	**$ODBC.Type**	4
**N**	**Valid**	31
	**Missing**	0
Central Tendency and Dispersion	**Mean**	5.77
	**Standard Deviation**	.990
	**Percentile 25**	6.00
	**Percentile 50**	6.00
	**Percentile 75**	6.00
